# Biallelic loss-of-function variants in *EXOC6B* are associated with impaired primary ciliogenesis and cause spondylo-epi-metaphyseal dysplasia with joint laxity type 3

**DOI:** 10.1002/humu.24478

**Published:** 2022-10-08

**Authors:** Pelin Ozlem Simsek-Kiper, Prince Jacob, Priyanka Upadhyai, Zihni Ekim Taşkıran, Vishal S. Guleria, Beren Karaosmanoglu, Gozde Imren, Rahsan Gocmen, Gandham S. Bhavani, Neethukrishna Kausthubham, Hitesh Shah, Gulen Eda Utine, Koray Boduroglu, Katta M. Girisha

**Affiliations:** 1 Department of Pediatric Genetics, Faculty of Medicine, Hacettepe University, Ankara, Turkey; 2 Department of Medical Genetics, Kasturba Medical College, Manipal, Manipal Academy of Higher Education, Manipal, India; 3 Department of Medical Genetics, Faculty of Medicine, Hacettepe University, Ankara, Turkey; 4 Department of Radiology, Faculty of Medicine, Hacettepe University, Ankara, Turkey; 5 Department of Pediatric Orthopaedics, Kasturba Medical College, Manipal, Manipal Academy of Higher Education, Manipal, India

**Keywords:** central nervous system anomalies, ciliopathy, EXOC6B, exocyst, joint dislocation, primary cilia, spondylo-epi-metaphyseal dysplasia with joint laxity type 3

## Abstract

Spondylo-epi-metaphyseal dysplasias with joint laxity, type 3 (SEMDJL3) is a genetic skeletal disorder characterized by multiple joint dislocations, caused by biallelic pathogenic variants in the EXOC6B gene. Only four individuals from two families have been reported to have this condition to date. The molecular pathogenesis related to primary ciliogenesis has not been enumerated in subjects with SEMDJL3. In this study, we report two additional affected individuals from unrelated families with biallelic pathogenic variants, c.2122+15447_2197-59588del and c.401T>G in *EXOC6B* identified by exome sequencing. One of the affected individuals had an intellectual disability and central nervous system anomalies, including hydrocephalus, hypoplastic mesencephalon, and thin corpus callosum. Using the fibroblast cell lines, we demonstrate the primary evidence for the abrogation of exocytosis in an individual with SEMDLJ3 leading to impaired primary ciliogenesis. Osteogenesis differentiation and pathways related to the extracellular matrix were also found to be reduced. Additionally, we provide a review of the clinical and molecular profile of all the mutation-proven patients reported hitherto, thereby further characterizing SEMDJL3. SEMDJL3 with biallelic pathogenic variants in *EXOC6B* might represent yet another ciliopathy with central nervous system involvement and joint dislocations.

## Introductions

Spondyloepimetaphyseal dysplasia with joint laxity, type 3 (SEMDJL3; MIM# 618395) is an autosomal recessive genetic skeletal disorder, caused by biallelic pathogenic variants in *EXOC6B* (MIM# 607880). Its characteristic features include delayed carpal ossification, gracile metacarpal and metatarsals, spine deformities, severe joint laxity, multiple joint dislocations, short stature, and delayed bone age. Two siblings of Indian origin were first reported with a homozygous truncating variant in *EXOC6B* (Girisha et al., 2016). Subsequently, two additional affected individuals, reported earlier, were molecularly diagnosed with an approximately 220 kb biallelic deletion spanning exons 9–20 of *EXOC6B* (Campos-Xavier et al., 2018; Spranger et al., 2006). These reports helped in defining a new clinical entity of SEMD in the Nosology and Classification of Genetic Skeletal Disorders under Group 20 “Dysplasias with multiple joint dislocations” (Mortier et al., 2019).

EXOC6B or Sec15B is a component of the exocyst that is a highly conserved, ~750 kDa octameric complex that mediates the initial tethering of secretory vesicles at the plasma membrane during exocytosis and membrane expansion (Hsu et al., 1996, 1998; Novick et al., 1980; Rivera-Molina & Toomre, 2013; TerBush & Novick, 1995). It comprises the subunits Sec3, Sec5, Sec6, Sec8, Sec10, Sec15, Exo70, and Exo84 in *Saccharomyces cerevisiae* or EXOC1–EXOC8 in mammals (Hsu et al., 1996). The assemblage of the exocyst complex at the plasma membrane is fostered by the direct interaction of Sec3/EXOC1 and Exo70/EXOC7 with phosphatidylinositol 4,5-biphosphate in the inner leaflet of the plasma membrane (Liu et al., 2007; Zhang et al., 2008). Sec15/EXOC6 interacts with Sec4, a Rab small guanosine triphosphatase (GTPases) in its GTP-bound state and anchors the exocyst complex to the secretory vesicles (Guo et al., 1999). In mammalian cells, the exocyst complex triggers membrane fusion likely via the EXOC7-mediated recruitment of the soluble *N*-ethylmaleimide-sensitive fusion attachment protein receptors (SNAREs) (Ahmed et al., 2019). The mammalian exocyst has been implicated in several processes fundamental to cell growth, proliferation, differentiation, and homeostasis, including primary ciliogenesis (Lipschutz, 2019; Martin-Urdiroz et al., 2016). Notably, Sec15/EXOC6 colocalized with the small GTPase Rab8, which is essential for cilia biogenesis, at the primary cilium, and Sec15 inhibition led to reduced primary ciliogenesis in hTERT-retinal pigmentary epithelial 1 (hTERT-RPE1) cell line (Feng et al., 2012).

In this study, we report on two unrelated probands from two families of different ethnicities with SEMDJL3, harboring biallelic disease-causing variants in *EXOC6B* (NM_015189.1). A detailed functional assessment of the variants was performed in subject-derived fibroblast cells, which demonstrated a complete absence of EXOC6B protein and a significant reduction in primary cilia length. These results provide the first evidence of the impairment of exocytosis potentially deregulating primary ciliogenesis in subjects with SEMDJL3. In addition, one of the patients exhibited clinical findings including intellectual disability and structural brain malformations such as hydrocephaly, hypoplastic mesencephalon, and thin corpus callosum, the findings that have not been reported in SEMDJL3 so far. Finally, we reviewed the clinical and radiological features in all individuals reported hitherto with molecular confirmation to further delineate the phenotypic spectrum of the disease.

## Materials And Methods

### 2.1 Editorial policies and ethical considerations

This study was approved by the Kasturba Medical College and Kasturba Hospital Institutional Ethics Committee (IEC:921/2018) and by the Hacettepe University Ethics Committee (GO 15-530/25). Written informed consent was obtained from the patients and family members for their participation in the study.

### 2.2 Clinical evaluation

The patients were subjected to detailed clinical evaluation by documenting their clinical features, photographs, radiological findings, and family history.

#### 2.2.1 Subjects and family history

**Figure 1:**
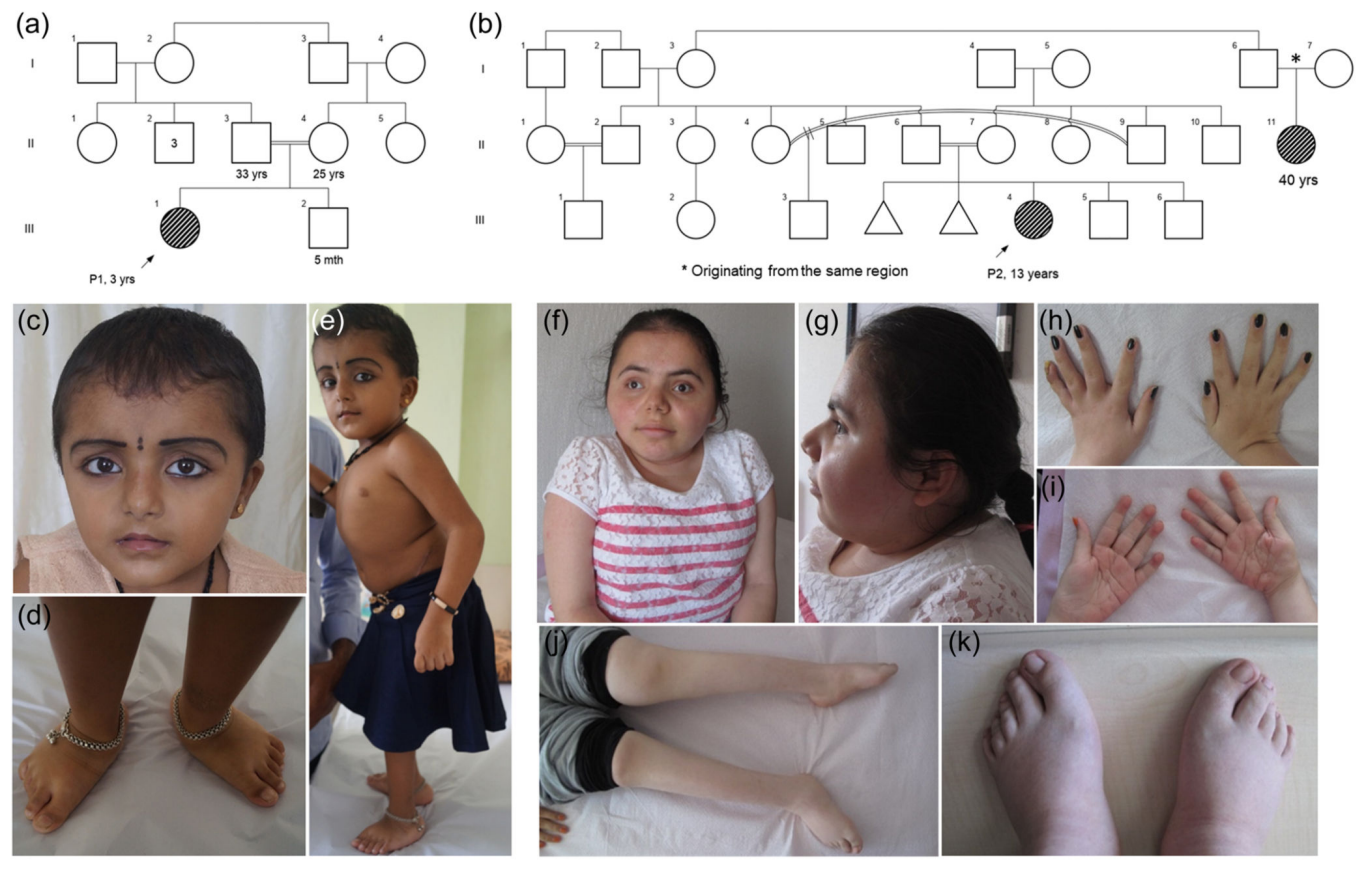
Pedigrees (a and b) show proband; P1 (III.1) and P2 (III.4) are born to consanguineously married couples. The other affected individual (III-11) is a 40-year-old female (b) with multiple joint dislocations, intellectual disability, scoliosis, and short stature; the parents of whom originate from the same region. Clinical evaluation was not performed for this patient. Clinical photographs of P1 at the age of 3 years show a broad forehead, overhanging columella, small chin, triangular face (c), bilateral flat feet, short 3rd, 4th, and 5th toes (d), and short neck and barrel-shaped chest (e). High and broad forehead, brachycephaly, short neck, small chin, high nasal bridge, a round face, barrel-shaped chest (f and g), deep palmar creases, bilateral 5th finger clinodactyly (h and i), genu deformity, short 3rd, 4th, and 5th toes, and prominent heels (j and k) are noted in P2 at age 13 years.

*Family 1:* Three-year-old girl (P1) of Indian ethnicity presented with difficulty in walking and inability to squat for 2 months before examination. She was born to a consanguineous couple (Figure 1a) at 9 months of gestation via normal vaginal delivery with a birth weight of 2.7 kg (+0.75 SD). She was admitted to the neonatal intensive care unit for 3 days in view of jaundice and was under phototherapy treatment for two nights. Her cognition and development milestones were age-appropriate except for a delay in gross motor skills. Two to three milliliters of blood samples were collected from the proband and family members for genetic testing.

*Family 2:* A 13-year-old girl (P2) of Turkish ethnicity presented with complaints of difficulty in walking and scoliosis. She could not walk independently and used a wheelchair. She was born to a healthy consanguineous couple (Figure 1b) at term via normal vaginal delivery with a birth weight of 1.75 kg (-3.60 SD). Birth length was not noted. She did not require neonatal intensive care unit admission. However, jaundice was noticed and she received phototherapy for 3 days. The prenatal history was unremarkable. The patient had a delay in motor and mental development milestones. She had head control after 12 months and could sit with or without support after 12 and 24 months of age, respectively. She started to talk after 2 years of age. She could attend primary school by 10 years of age. She could read and write, but required special education. Hydrocephalus and bilateral developmental hip dysplasia were diagnosed when she was 3 and 6 years old, respectively. However, no intervention was performed for either of them.

### 2.3 Exome sequencing (ES) and copy-number variant (CNV) analysis

ES in P1 was performed using the Agilent V6 capture kit based on the method previously reported (Kaur et al., 2021). A cascade of filtering steps was performed to prioritize and identify the variant likely to cause the disease (Supporting Information: Table S1). Pathogenicity assessment was performed based on various parameters, including genomic location, clinical correlation, OMIM phenotypes, patterns of inheritance, and scores of multiple in silico prediction tools. The genotype quality and read depth of variants were also assessed.

CNV analysis was performed from ES data of proband (P1) using the ExomeDepth tool (Plagnol et al., 2012). Using an in-house database of ES data, CNV calling was performed against reference samples (63 reference samples) of the Agilent V6 capture kit (Kausthubham et al., 2021). A total of 53 CNVs (31 deletions and 22 duplications) were called. CNVs implicated in the pathogenesis of skeletal dysplasia were filtered and manual inspection was performed using Integrated Genomic Viewer (IGV). True CNV calls were then correlated with the existing literature for clinical features, inheritance patterns, and disease mechanisms, followed by evaluating the CNV frequency in the control population databases (DGV), another database for interpretation of genomic variations (DECIPHER), and ClinVar. Guidelines issued by the American College of Medical Genetics and Genomics (ACMG) and the Clinical Genome Resource (ClinGen) were followed for the interpretation of constitutional CNVs (Riggs et al., 2021). ES in P2 was performed by using an Ampliseq Exome RDY kit with Ion Proton Sequencer. Homozygosity mapping and variant filtering were done according to the previous report (Utine et al., 2017).

### 2.4 Primer walking

To investigate the breakpoints of the deletion in the genomic region of *EXOC6B* (NC_000002.12) in P1, the genomic region between exons 19 and 21 of *EXOC6B* was divided into smaller sections. The genomic region was further evaluated by PCR and sequencing in the patient, her parents, unaffected sibling, and an unrelated control. To this end, a set of 46 primer pairs were designed using NCBI Primer-BLAST (https://www.ncbi.nlm.nih.gov/tools/primer-blast/).

### 2.5 Cell culture

Dermal fibroblasts were obtained from P1 and P2. Fibroblast cell lines were cultured in Dulbecco’s modified Eagle’s media (4.5 g/L glucose, cat. no. 10566016; Invitrogen) supplemented with 10% heat-inactivated fetal bovine serum (cat. no. RM10409; HiMedia), 100 U/ml penicillin, and 100 μg/ml streptomycin (cat. no. A018; HiMedia) and were propagated in a humidified atmosphere (37°C, 5% CO_2_).

### 2.6 RNA isolation and analyses

#### 2.6.1 Patient 1

Total RNA was extracted from fibroblasts and blood samples using TRIzol reagent (cat. no. 15596026; Invitrogen). The total RNA content and purity in each sample were assessed by absorbance at 260 nm and A260/280 ratio, respectively. RNA was reverse transcribed using Superscript IV Vilo mastermix™ (cat. no. 11756050; Invitrogen) according to the manufacturer’s protocol. Reverse transcription-PCR (RT-PCR) was performed using GoTaq Green Master Mix (cat. no. M7122; Promega). RT-quantitative real-time PCR (RT-qPCR) was carried out using StepOne (Applied Biosystems/Thermo Fisher) with a final reaction volume of 10 μl. All reactions were prepared with 5 μl of 2× PowerUP™ SYBR™ Green Master Mix (cat. no. A25776; Applied Biosystems), and run in duplicates for each of three independent replicates. The messenger RNA (mRNA) levels for target genes were normalized to *GAPDH*. Relative quantification was carried out using the 2^−ΔΔ*C*_T_^ method. To discern the aberrant splicing products the PCR products amplified were evaluated by Sanger sequencing. The oligonucleotides used in RT-PCR and RT-qPCR are listed in Supporting Information: Table S2.

#### 2.6.2 Patient 2

Total RNA was isolated from passage 3 donor fibroblast cells with TRIzol reagent (cat. no. 15596026; Invitrogen). Concentration and purity were quantified by spectrophotometer (NanoDrop 2000; Thermo Fisher Scientific). All samples were treated with RNase-free DNase I (cat. no. 4716728001; Roche Diagnostics). Complementary DNA (cDNA) synthesis from total RNA was performed using the High Capacity cDNA Reverse Transcription kit (cat. no. 4374966; Applied Biosystems) according to the manufacturer’s instructions. Primers specific to *EXOC6B* and LightCycler 480 SYBR Green I Master (cat. no. 04707516001) were used for amplification. Quantitative real-time RT-PCR was performed with LightCycler 480 Real-Time PCR System (Roche Diagnostics). *ACTB* was used as a housekeeping gene. Each sample was analyzed in triplicate reactions. Relative levels of mRNA gene expression were calculated using the 2^−ΔΔ*C*_T_^ method.

#### 2.7 Immunofluorescence (IF) and fluorescence microscopy

Fibroblast cells were seeded on glass coverslips and propagated till 90% confluence. Cells were fixed in 4% (wt/vol) paraformaldehyde (PFA), permeabilized in 0.2% Triton X-100, blocked in 5% normal goat serum, and incubated overnight at 4°C with primary antibodies diluted in blocking solution. Subsequently, they were incubated in the following secondary antibodies: Alexa Fluor 488 goat anti-rabbit and Alexa Fluor 568 goat anti-mouse IgG (cat. no. A11034 and cat. no. A11031, respectively; Molecular Probes) diluted at 1:500 for 2 h at room temperature (RT). Nuclei were stained using DAPI (4’,6-diamidino-2-phenylindole; cat. no. D1306; Invitrogen) and cells were mounted in Prolong Diamond Antifade mountant (cat. no. P36961; Invitrogen). Images were acquired using an inverted fluorescence microscope with LD Plan-Neofluar 63×X/0.75 Corr Ph2 oil immersion objective and Axiocam 503 CCD camera (Axiovert A1 FL; Zeiss) and processed using Fiji (imagej.net/Fiji).

#### 2.8 Evaluation of primary cilia

To induce ciliogenesis and evaluate primary cilia, fibroblasts were serum starved as described earlier before immunostaining (Upadhyai et al., 2020). Primary cilia were ascertained by dual labeling with antibodies against acetylated α-tubulin and Arl13B. Their length was determined manually by tracing them using Fiji (imagej.net/Fiji). Primary cilia lengths are represented in micrometer (μm).

#### 2.9 Immunoblotting (IB)

Fibroblasts were harvested in ice-cold radioimmunoprecipitation assay buffer (cat. no. 20-188; Merck Millipore) and supplemented with a 1× mini protease inhibitor cocktail (cat. no. 11836153001; Roche) on ice for 15min. Cell debris was removed by centrifugation at 20,000g for 30 min at 4°C. Protein extracts (50 μg) were separated on SDS-PAGE and transferred to polyvinylidene fluoride membranes (cat. no. 1620177; Bio-Rad) using a semidry transfer system (cat. no. PB0010; Invitrogen). Membranes were blocked by incubation in TBST buffer (TBS with 0.1% Tween-20) supplemented with 5% blotting-grade blocker (cat. no. 1706404; Bio-Rad) for 1h at RT. Followed by incubation in primary antibodies diluted in the blocking solution overnight at 4°C. Membranes were incubated with the following secondary anti-bodies, HRP-conjugated anti-rabbit (cat. no. 1706515) and anti-mouse (cat. no. 1706516) from Bio-Rad diluted at 1:10,000 for 2 h at RT. Immunoreaction was detected using SuperSignal™ West Pico Plus chemiluminescent substrate (cat. no. 34577; Thermo Fisher Scientific). IBs were digitally detected using iBright FL1500 Imaging System (Invitrogen).

#### 2.10 Primary antibodies

Antiacetylated α-tubulin (mouse monoclonal, cat. no. T7451; Sigma-Aldrich; IF: 1:4000), anti-Arl13B (rabbit polyclonal, cat. no. 17711-1-AP, ProteinTech; IF: 1:3000), anti-EXOC6B (rabbit polyclonal, cat. no. NBP2-56692, Novus Biologicals; IB: 1:200) and anti-β-actin (mouse monoclonal, cat. no. A1978; Sigma-Aldrich; IB: 1:5000).

#### 2.11 Osteogenic differentiation assay

Osteogenic differentiation assay was performed with StemPro™ Osteogenesis Differentiation kit (cat. no. A1007201; Thermo Fisher Scientific) according to the manufacturer’s instructions with both the dermal fibroblasts of the patient (Patient 2) and a healthy donor (ATCC no: PCS-201-012™; lot no: 63792061). After 21-day differentiation, cells were fixated with 3.7% PFA, washed with PBS, stained with Alizarin Red S, and visualized under a light microscope.

#### 2.12 Total mRNA sequencing

SENSE Library kit (cat. no. 001.24; Lexogen) was used for library preparation. Following the library preparation, clonal amplification was performed with Ion PI Hi-Q T2 200 kit (cat. no. A26434; Thermo Fisher Scientific) and sequencing was conducted on the Ion Proton Instrument (Thermo Fisher Scientific) by using the Ion PI Hi-Q Sequencing 200 kit (cat. no. A26433; Thermo Fisher Scientific). All samples were analyzed in duplicates. For data analysis, the adaptors were trimmed from the raw data following which the GC content was calculated. The reference genome was built using Bowtie and paired-end clean reads were aligned using TopHat. TPM (transcripts per kilobase million) were calculated after read numbers were mapped to each gene. Differential expression analysis was performed with DESeqR package. Genes with an adjusted *p* < 0.05 were assigned as differentially expressed. Gene Ontology enrichment analysis was implemented by the GOSeq R package with corrected *p* < 0.05. Pathway and process enrichment analysis was done in RaNA-Seq. (https://ranaseq.eu/ Carlos Prieto and David Barrios. RaNA-Seq: Interactive RNA-Seq analysis from FASTQ files to functional analysis. Bioinformatics, btz854, 10.1093/bioinformatics/btz854)

#### 2.13 Statistical analyses

Data analysis was carried out by GraphPad Prism (v9.0) (www.graphpad.com/scientific-software/prism/). All experiments were performed in three independent replicates and statistical analyses were performed using one-way or two-way analysis of variance (ANOVA), as applicable, followed by Tukey’s post hoc test for multiple comparison; *p*<0.05 was considered statistically significant (*p<0.05; **p<0.01; ***p < 0.001; ****p < 0.0001). Primary cilia frequency and length values are shown as mean ± standard error of the mean (SEM).

## Results

### 3.1 Clinical description (Families 1 and 2)

On clinical examination of Proband 1 (P1), at 3 years of age, the height was 85 cm (−2.8 SDs), and weight was 12 kg (−1.5 SDs). Her facial features include a broad forehead, overhanging columella, small chin, and triangular face. Bilateral flat feet, short 3rd, 4th, and 5th toes, short neck, and barrel-shaped chest were also remarkable (Figure 1c–e). The ophthalmological evaluation showed no signs of ocular defects.

Physical examination of Proband 2 (P2) at the age of 13 years revealed a body height of 116 cm (−5.83 SDs), body weight 34 kg (−1.79 SDs), head circumference 51.5 cm (−2.37 SDs), and arm span of 116 cm. Body mass index (BMI) was 25 (1.5 SD). Facial features including brachycephaly, high and broad forehead, small chin, high nasal bridge, and a round face were noted (Figure 1f,g). She had nystagmus, short neck, kyphoscoliosis, barrel-shaped chest, stria, bilateral 5th finger clinodactyly, short 3rd, 4th, and 5th toes, deep palmar creases, genu valga deformity, and prominent heels. Fingers were long and slender with distal tapering. Hypermobile joints, yet with restricted mobility of the large joints including knees and elbows were noted as well (Figure 1h–k). Psychometric assessment with WISC-R revealed mild intellectual disability. The ophthalmological evaluation revealed mild myopia. Audiologic assessment was normal. Pulmonary function test revealed findings compatible with airway obstruction (forced vital capacity [FVC]: 53%; forced expiratory volume in 1 s [FEV1]: 46%; FEV1%: 78%; Forced expiratory flow at 25 and 75% of the pulmonary volume [FEF25–75]: 26%; vital capacity [VC]: 48%). Bone mineral densitometry of the lumbar spine (L1–L4) revealed a *z*-score of −3.3. The serum levels of calcium, phosphate, alkaline phosphatase, vitamin D, and parathyroid hormone were all normal. On her recent evaluation at the age of 20 years, body height was 123 cm (<3rd centile, 3rd centile: 151 cm), body weight was 39 kg (<3rd centile, 3rd centile: 45 kg), and head circumference was 52m. BMI was 25.7 (BMI SD score: 1.06). The patient had menarche by 17 years of age. Pulmonary function test revealed moderate airway restriction (FVC: 49%; FEV1: 46%; FEV1%: 80%; FEF25–75: 17%; VC: 53%). Weight, height, and BMI charts of Patient 1 (at 3 years of age) and Patient 2 (at 13 years of age) are shown in Supporting Information: Figure S1. The carrier parents were phenotypically normal in both families and we did not have access to their fibroblasts.

### 3.2 Radiological description (Families 1 and 2)

**Figure 2:**
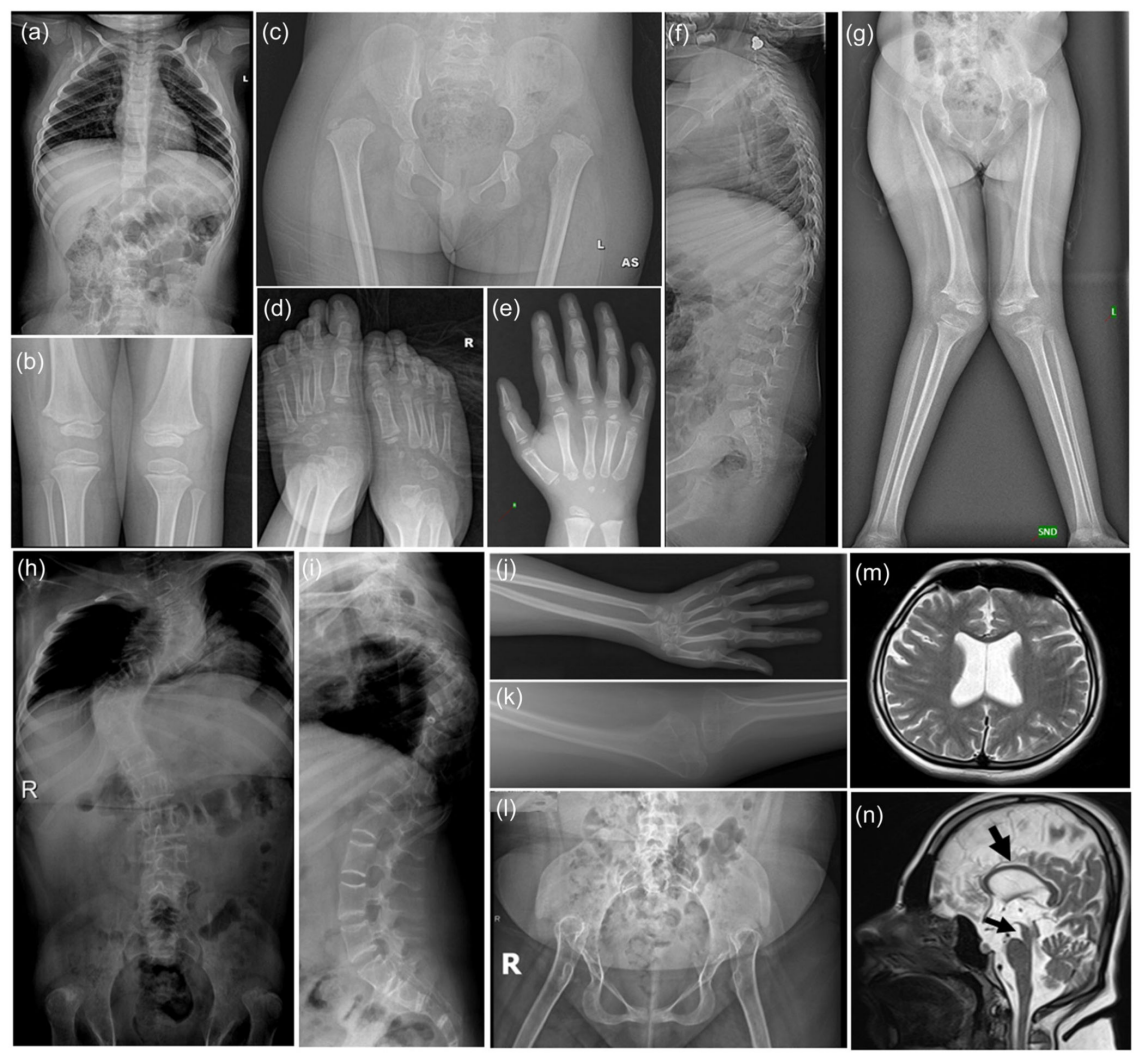
Radiographs of Patient 1 (P1) at age 3 years show narrow interpedicular distance in lumbar vertebrae, slender ribs (a), small epiphyses and irregular metaphyses at the knee (b). Bilateral hip dislocation, small femoral epiphyses and narrow acetabulum are evident (c). Slender metatarsals/leptodactyly in hands and feet and delayed carpal bone ossification in hands are noted (d and e). She has exaggerated lumbar lordosis and irregular vertebral end plates (f). Bilateral dislocation of knees and genu valgum is observed in proband at the age of 5 years and 7 months (g). Radiographs of Patient 2 (P2) at 13 years of age show severe thoracic kyphoscoliosis, slender ribs, increased lumbar vertebra body heights, and irregular and sclerosing endplates (h and i). Radiographs of limbs (j) show diffuse osteopenia and slender long and short tubular bones. Note that the metacarpals are leptodactylic and knee (k) and hip joints (l) are dislocated. Bilateral shallow acetabular fossa, short iliac wings, metaphyseal widening and epiphyseal irregularity, enlarged distal phalangeal tufts, and distal radioulnar subluxation are also observed (l). Brain magnetic resonance imaging (m and n) of P2 at 13 years of age (axial and sagittal T2-weighted brain magnetic resonance images) revealed hydrocephalus, brachycephaly, thin corpus callosum (thick arrow), and hypoplastic mesencephalon (thin arrow).

Radiological survey at age 3 years of Proband 1 revealed small epiphyses, leptodactyly with delayed carpal bone ossification in hands, and narrow interpedicular distance in lumbar vertebrae with exaggerated lumbar lordosis and irregular vertebral end plates. Slender ribs, small epiphyses, and irregular metaphysis were observed in the knees, whereas bilateral hip dislocation, small epiphyses of the femur, and irregular acetabulum were observed in the pelvis and hips. Slender metatarsals were noted in the feet. Genu valgum and dislocation of knees were observed at the age of 5 years and 7 months (Figure 2a–g).

Radiographs of Proband 2 (P2) at age of 13 years revealed severe kyphoscoliosis, diffuse osteopenia, and slender long and short tubular bones (Figure 2h–k). Cranial magnetic resonance imaging revealed a thin corpus callosum, hypoplasia of the mesencephalon, and hydrocephaly (Figure 2m,n).

### 3.3 Identification and molecular analysis of pathogenic variants in *EXOC6B*

#### 3.3.1 Family 1

**Figure 3:**
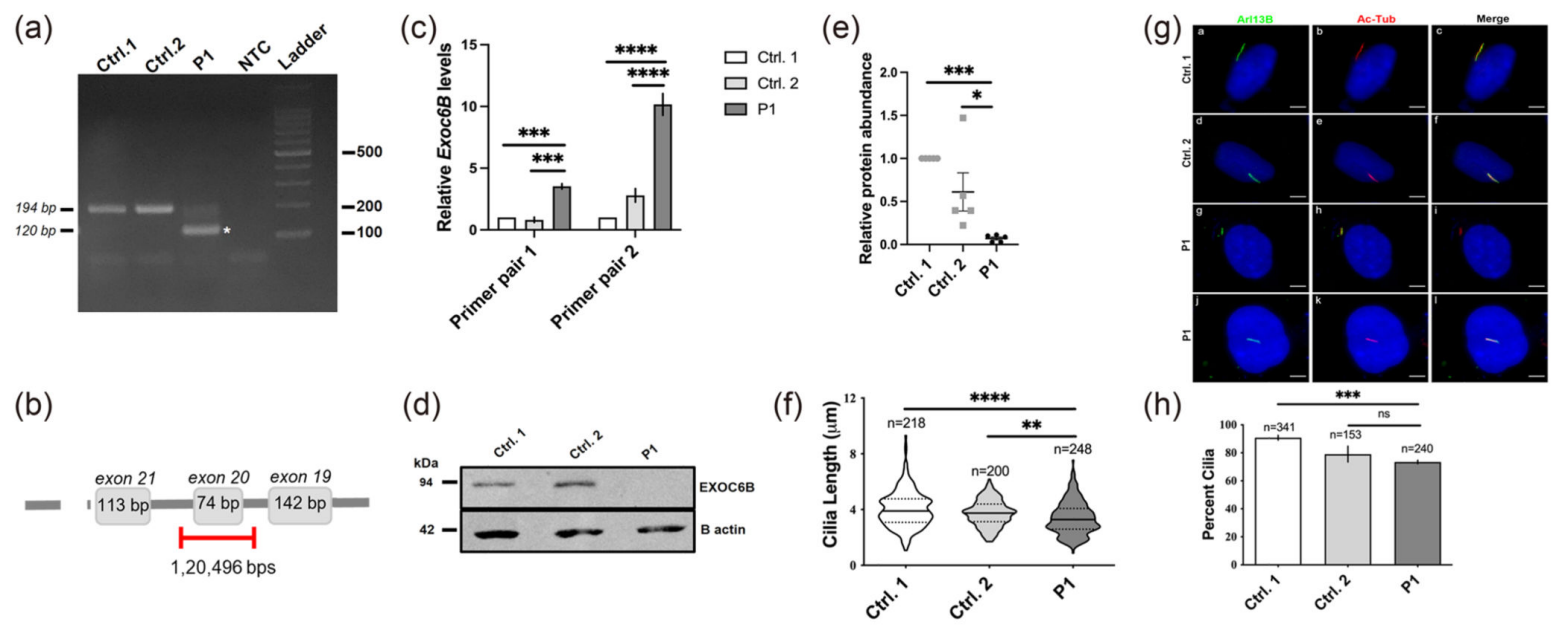
EXOC6B mRNA and protein analysis in P1. (a) RT-PCR analyses in fibroblast-derived cDNA from P1 and two healthy controls. The c.2122+15691_2197-59634del in EXOC6B results in a shorter PCR product (120 bp) that is indicated with a white asterisk (*) owing to the deletion of exon 20 (74 bp) in P1. The size of this amplicon is 194 bp and is detected in control cDNA. (b) Schematic representation of the c. 2122+15447_2197-59588del in EXOC6B in P1, which spans 120,496bp removing exon 20 and its flanking intronic regions. (c) Relative quantification of EXOC6B mRNA levels by RT-qPCR using two distinct primer pairs 1 and 2 revealed significantly elevated EXOC6B transcript levels in P1; levels are normalized to GAPDH. Bars represent the mean ± SE of three independent experiments (*n* = 3), each performed in duplicate. Statistical difference was evaluated by two-way ANOVA, followed by Tukey’s post hoc analyses: ***p < 0.001; ****p < 0.0001. (d) Immunoblot analyses of EXOC6B protein (molecular mass ~94 kDa) in whole-cell lysates obtained from fibroblasts of the P1 and two controls revealed a complete absence of EXOC6B in P1; β-actin was used as a loading control. (e) Quantitative immunoblot analysis with β-actin was used for normalization; horizontal lines represent the mean of immunoblot signal analyses performed five times ((*n* = 5). Error bars indicate SE; statistical analyses were performed by one-way ANOVA, followed by Tukey’s post hoc analyses: *p < 0.05; ***p < 0.001. (f) Quantification of primary cilia length in subject and control fibroblast cell lines revealed primary cilia length was significantly reduced in P1 versus both controls; one-way ANOVA followed by Tukey’s post hoc analyses: **p < 0.01, ****p < 0.0001; number of nuclei evaluated are as follows: (*n* = 218 (Ctrl.1), (*n* = 200 (Ctrl. 2), and (*n* = 248 (P1). (g) Absence of EXOC6B affects primary ciliogenesis in fibroblasts from P1. (a–1) Primary cilia were dual immunolabelled in fibroblasts from the affected subject P1 and healthy controls with antibodies against Arl13B (green) and acetylated α-tubulin (red) that label the ciliary axoneme. Nuclei were labelled with DAPI (blue). Scale bar = 5 μm (white line). (h) Quantification of prevalence in subject and control fibroblast cell lines showed that the frequency of primary cilia was significantly reduced in P1 when compared to Ctrl. 1 but not Ctrl. 2; one-way ANOVA followed by Tukey’s post hoc analyses: ***p < .001, ns: nonsignificant; number of nuclei evaluated are as follows: (*n* = 341 (Ctrl. 1), (*n* = 153 (Ctrl. 2), and (*n* = 240 (P1), and protein analysis in P1. ANOVA, analysis of variance; cDNA, complementary DNA; Ctrl.1, control 1; Ctrl.2, control 2; GAPDH, glyceraldehyde 3-phosphate dehydrogenase; mRNA, messenger RNA; NTC, no template control; RT-qPCR, RT-quantitative real-time PCR; RT-PCR, reverse transcription-PCR; SE, standard error.

Analysis of the ES data from P1 in Family 1 did not reveal any potential disease-causing variants likely to be disease causing. However, CNV analysis by ExomeDepth tool (Plagnol et al., 2012) suggested a likely 72 bp homozygous deletion of exon 20 in *EXOC6B* (Supporting Information: Table S3) in the patient, which was corroborated upon manual inspection of the genomic region in IGV (Supporting Information: Figure S2). RT-PCR using cDNA derived from fibroblast and blood, followed by sequencing further confirmed the homozygous deletion of exon 20 in P1 (Figure 3a). We resorted to a primer walking approach to delineate the breakpoints of the deletion in P1 genomic DNA and identified a homozygous deletion of ~120kb (12,0496 bp) in the proband, NM_015189.1: c.2122+15447_2197-59588del, p.(Gln708Profs*16), which encompasses the entire exon 20 and flanking intronic regions (chr2:72470904–72591411; NC_000002.12) of *EXOC6B* (Figure 3b and Supporting Information: Figure S3). We validated this deletion through cDNA sequencing in the proband and no other variations were identified in the complete cDNA. Through segregation analysis, we confirmed that this deletion was present in the heterozygous state of her parents and was not observed in her unaffected brother (Supporting Information: Figure S4). This variant was not observed in any individual in the gnomAD population database and our in-house database of 1455 exomes.

To assess the molecular effects of the c.2122+15447_2197-59588del variant in *EXOC6B* on mRNA processing in P1, we evaluated the *EXOC6B* mRNA levels and unexpectedly observed it to be significantly elevated compared to two unrelated control fibroblast cell lines (Figure 3c). In contrast, EXOC6B protein was absent in fibroblast-derived cell lysates (Figure 3d,e). Primary cilia observed in control cells ranged from 3.74 ± 0.07 to 4.03 ± 0.09 μm in length; however, the ciliary length was reduced to 3.32 ± 0.11 μm in affected cells (Figure 3f). Given the role of EXOC6/Sec15 in ciliogenesis, we evaluated primary cilia frequencies in the subject and control-derived fibroblasts and observed significant shortening of primary cilia length in P1 compared to controls (Figure 3g). Minor variability was noted in primary cilia prevalence in P1, although it did not appear to be conclusively altered when compared to control cell lines (Figure 3h).

#### 3.3.2 Family 2

**Figure 4:**
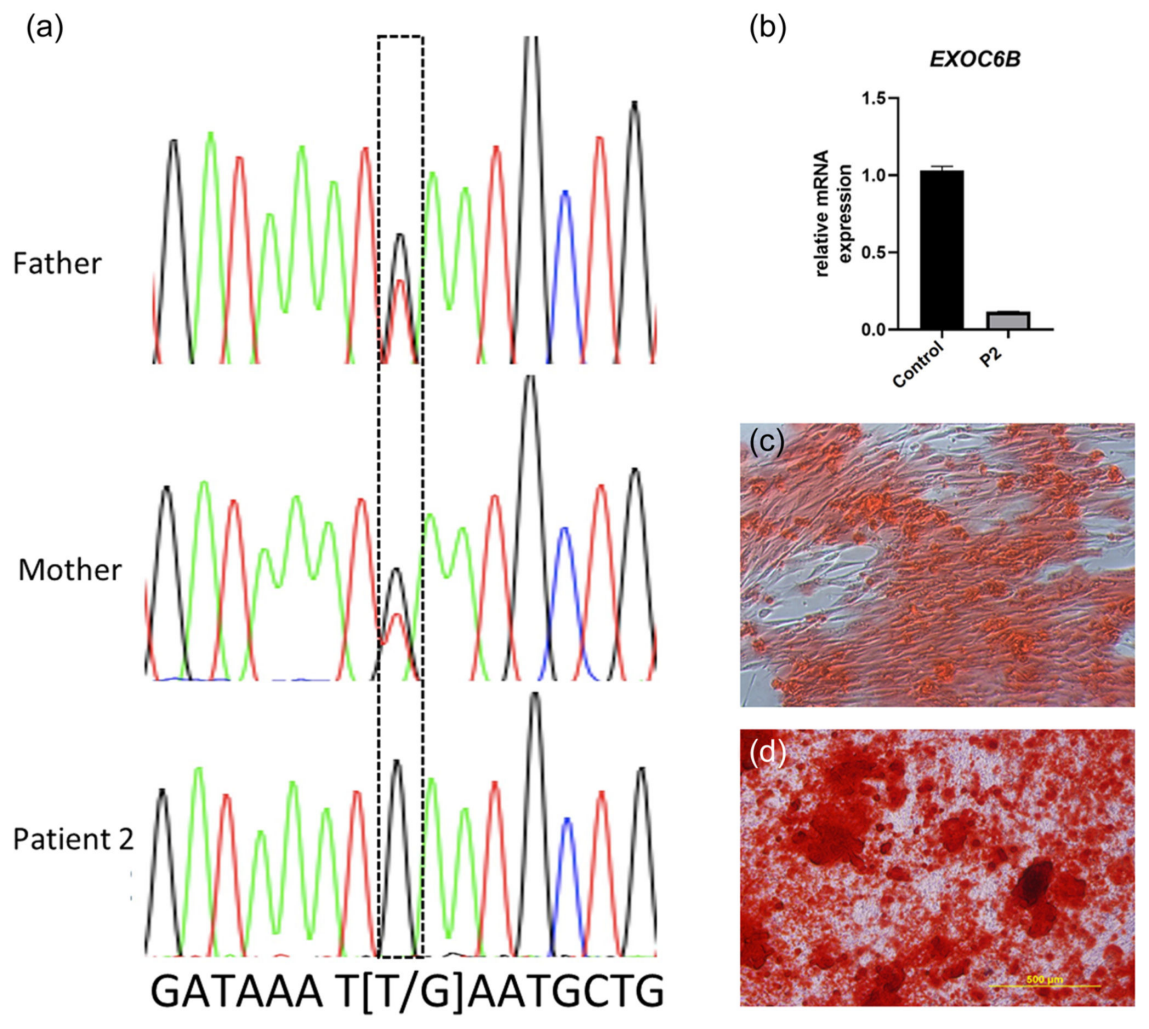
Patient (P2) is homozygous for c.401T>G p.(Leu134Ter) and the parents are heterozygous for this variant. The variant detected via exome sequencing was validated with Sanger sequencing in Patient 2 (a). EXOC6B expression was found to be decreased in dermal fibroblasts of Patient 2 (P2) as compared to that of the healthy control (b). To determine the osteogenic differentiation potential of the dermal fibroblast of P2, Alizarin Red S staining was used, which showed that the osteogenic differentiation of P2 dermal fibroblast was decreased (c) compared to that of a healthy donor’s dermal fibroblast (d). mRNA, messenger RNA.

In P2, ES analysis revealed 11 homozygous regions in 10 different chromosomes. In these regions, there was only one variant located in exon 4, which was probably responsible for the patient’s phenotype: NM_015189.1: c.401T>G p.(Leu134Ter) *EXOC6B* (Figure 4a).

In P2, the expression of *EXOC6B* was found to be decreased in dermal fibroblasts (Figure 4b). We also performed RNA sequencing in fibroblasts of P2. It was observed that the pathways related to the extracellular matrix were decreased in the patient’s fibroblasts (Supporting Information: Figure S5). The osteogenic differentiation potential of the dermal fibroblast of the P2 was compared with a healthy donor dermal fibroblast. According to the Alizarin Red S staining, it was observed that the osteogenic differentiation of the P2 dermal fibroblasts was decreased (Figure 4c,d).

The novel *EXOC6B* variants identified in P1 and P2 in this study have been submitted to ClinVar with the submission and accession IDs of SUB10588140 SCV001984873, and SUB10986427 and SCV002062066, respectively. *EXOC6B* variants causing SEMDJL3 along with the variant identified in the present study are shown in Supporting Information: Figure S6.

## Discussion

In this study, we report on the clinical and mutational findings of two affected individuals with SEMDJL3 from two unrelated families and we demonstrate that biallelic loss-of-function variants in *EXOC6B* are associated with impaired primary ciliogenesis.

The coordinated intracellular transport between membrane-bound compartments is fundamental to cell growth, proliferation, signaling, differentiation, and homeostasis. The delivery of cellular cargo such as proteins and lipids to appropriate compartments or the plasma membrane involves the docking of cargo-laden transport vesicles at target membranes and their subsequent fusion. Membrane trafficking is mediated by the synchronized action of vesicle coat proteins, multisubunit tethering complexes (MTCs), SNAREs, Sec1/Munc18 (SM) proteins, and Rab GTPases (Cai et al., 2007; Dubuke & Munson, 2016). MTCs promote the initial interaction between a vesicle and its target membrane and are critical for the delivery of cargo to specific cellular destinations (Bröcker et al., 2010; Dubuke & Munson, 2016; Yu & Hughson, 2010). The exocyst, along with Dsl1, Golgi-associated retrograde protein, conserved oligomeric Golgi complexes are categorized as CATCHR (complexes associated with tethering containing helical rods) family of MTCs owing to the structural similarities in their subunit organization (Dubuke & Munson, 2016; Yu & Hughson, 2010). Notably, the exocyst components have been detected at the trans-Golgi, recycling endosomes, and plasma membrane (Ang et al., 2004; Yeaman et al., 2001). While they can independently occur as free subunits or subcomplexes, their dynamic assembly into the complete octameric state is essential to promote membrane fusion (Ahmed et al., 2019).

The exocyst plays essential roles in the secretory pathway as evidenced by the early embryonic lethality observed in mice following the knockout of several members of the exocyst complex. *Exoc4/Sec8^-/-^* mice initiate gastrulation but die shortly afterward (Friedrich et al., 1997), *Exoc1/Sec3*-null mice undergo peri-implantation lethality (Mizuno et al., 2015), and the conditional ablation of *Exoc5/Sec10* leads to bilateral hydronephrosis and complete anuria in newborn mice leading to death shortly after (Fogelgren et al., 2015). Strikingly, the abrogation of Sec15/EXOC6 causes impaired erythroid iron assimilation and no other defects in hemoglobin-deficit (*hbd*) mice (Garrick & Garrick, 2007; Lim et al., 2005; White et al., 2005). Sec15 has been shown to play a role in late erythroid differentiation in the bone marrow (Bloom & Simon-Stoos, 1997). Both Sec15/EXOC6 and Sec15B/EXOC6B are widely expressed in mice tissues (Lim et al., 2005), suggesting that they may potentially play largely redundant roles in most tissues.

The exocyst function is important for ciliogenesis. Consistent with this, pathogenic variants in *EXOC2* cause a severe neurodevelopmental disorder with dysmorphic facies and cerebellar hypoplasia (MIM# 619306) leading to reduced exocytosis and defective Arl13B localization to the primary cilia in patient cells (Van Bergen et al., 2020). In addition mutations in *EXOC8* and *EXOC4* have been implicated in Joubert and Meckel-Gruber syndromes, respectively (Dixon-Salazar et al., 2012; Shaheen et al., 2013). Further, the disruption of EXOC5/Sec10 has been shown to cause deregulated primary cilia biogenesis in Madin–Darby canine kidney cells (Zuo, Guo, & Lipschutz, 2009). Several exocyst components and their interacting proteins localize to the primary cilium, for example, Sec5/EXOC2, Sec8/EXOC4, Sec10/EXOC5, Sec15/EXOC6A, and Rab10 (Feng et al., 2012; Rogers et al., 2004; Seixas et al., 2016). Moreover, some exocyst members interact with ciliary proteins; Sec5 and Sec8 directly interact with Arl13B (Seixas et al., 2016) and Sec10 directly interacts with the ciliary proteins IFT88 and polycystin-2 (Fogelgren et al., 2011; Zuo et al., 2009). It is noteworthy that both knockdown and overexpression of Sec15/EXOC6A cause shortening of primary cilia length in hTERT-RPE1 cells (Feng et al., 2012). Congruent with this, we observe significantly shortened primary cilia in P1-derived fibroblast cells. However, the reduction in primary cilia frequency in cells derived from P1 appeared moderate and variable compared to control cells. Our study is limited by the lack of testing P2 and not using multiple controls. The significance of these findings would need testing further patients with SEMDJL3.

Two previous studies reported pathogenic variants in EXOC6B in patients with SEMDJL3 (Campos-Xavier et al., 2018; Girisha et al., 2016; Spranger et al., 2006). Here, we report on two unrelated probands from two families of distinct ethnicities. All affected subjects had common phenotypes including spine anomalies, epiphyseal and metaphyseal dysplasia, multiple joint dislocations, gracile metacarpals, and metatarsals along with long slender fingers (Table 1), which defines this clinical entity. The absence of other syndromic clinical features such as facial dysmorphism, cleft palate, airway obstruction, congenital heart disease, kyphoscoliosis at birth, and talipes equinovarus distinguishes this clinical entity from other related SEMDs with joint laxity (MIM# 271640, 603546, and 608684) (Girisha et al., 2016). Interestingly, P2 has additional findings such as developmental delay and central nervous system anomalies. Although a formal psychometric assessment could not be performed, she had a delay in developmental milestones for which she required special education from the age of 9 years. She could recognize colors but could not recognize time on the clock, could not do simple mathematical calculations, and was dependent on her parents in her daily self-care. *EXOC6B* variants have previously been suggested in the genetic etiology of developmental delay (Evers et al., 2014; Girisha et al., 2016). In addition, low-normal intelligence was reported in one of the two sisters originally described by Spranger et al. (2006) who was later found to have biallelic *EXOC6B* deletion (Campos-Xavier et al., 2018). However, the developmental delay has not been considered among one of the clinical features of SEMDJL3 so far.

The clinical and neuroimaging findings of P2 might suggest a ciliopathy, a pleiotropic group of disorders with overlapping phenotypes, resulting from dysfunctions of cilia, cellular organelles with motile, and sensory and/or signalling functions (Baker & Beales, 2009; Mitchison & Valente, 2016). Various structural brain abnormalities including hydrocephalus, corpus callosum abnormalities, and midbrain anomalies have previously been reported in ciliopathies (Andreu-Cervera et al., 2021; Poretti et al., 2017; Shaheen et al., 2013). However, additional patients and functional studies are required before such a conclusion could be made.

The proband in Family 1 is homozygous for an ~120 kb deletion in *EXOC6B* that removes the entire exon 20 and flanking intronic regions leading to complete depletion of EXOC6B protein yet with significantly increased levels of mRNA. This region appeared to contain putative regulatory sequences including enhancers, transcription factor and CTCF-binding motifs, and a noncoding *EXOC6B* transcript (Supporting Information: Figure S7). Consequently, we surmise that its complete loss in P1 likely results in aberrant *EXOC6B* mRNA upregulation. However, the resultant mutant *EXOC6B* transcript is likely unstable and degraded. Intronic regions are increasingly understood to play important transcription regulatory roles in modulating gene expression levels and splicing (Bryen et al., 2019; Petibon et al., 2016), especially with regard to highly expressed and largely ubiquitous genes (Rose, 2018). Negative regulation of transcription by intronic regions has been observed for genes such as *PCNA* and *BRCA1* wherein intron deletion led to upregulation of mRNA expression (Alder et al., 1992; Chang et al., 1990; Suen & Goss, 2001). Further deletions of intronic and/or exonic regions harboring regulatory activity have been demonstrated to underlie several Mendelian disorders (Allou et al., 2021; Tayebi et al., 2014; Yamamoto-Shimojima et al., 2021). Alternatively increased *EXOC6B* mRNA despite the absence of protein may plausibly be accounted for by a negative feedback loop mechanism wherein deletion of its regulatory regions abrogates its translation or deregulates downstream components that in turn may stimulate its mRNA transcription. The proband in Family 2 is homozygous for the pathogenic novel variant in exon 4 of *EXOC6B*, c.401T>G p.(Leu134Ter). The parents were heterozygous for the same variant, confirming the homozygosity in P2. According to the ACMG criteria (Richards et al., 2015), the detected variant was predicted to be “pathogenic.” The expression of EXOC6B was found to be decreased in dermal fibroblasts, indicating that the mutant transcript was most likely to be degraded by nonsense-mediated decay. The RT-qPCR experiments for P1 and P2 were performed at two different centers; we could not organize a similar methodology and hence M and CV values of the housekeeping genes (*GAPDH* and *ACTB*) could not be calculated. Again, using more housekeeping genes in our experiments would have been better.

Another finding in this study was the observation of the decreased osteogenic differentiation of the dermal fibroblasts of P2 compared to that of the healthy control. In addition, RNA sequencing in the fibroblast sample of Patient 2 revealed that pathways related to the extracellular matrix were also decreased. *EXOC6B* is widely expressed in different human tissues including testis, ovary, central nervous system with the highest expression and skeletal muscle, pancreas, fetal liver, and fetal brain with little or no expression (Nagase et al., 1998). The decrease in ECM elements and collagen matrix in mRNA levels, as well as delay/reduction in osteogenic differentiation, may be the cellular consequences of EXOC6B loss of function. However, additional experiments are required to prove this mechanism.

In conclusion, we report on two affected individuals from two families with pathogenic variants in *EXOC6B* with SEMD and multiple joint dislocations. Taken together with existing reports, our studies provide initial evidence that depletion of EXOC6B likely impairs exocytosis and abrogates primary ciliogenesis in subjects with SEMDJL3.

## Supplementary Material

Supplementary file

## Figures and Tables

**Table 1:** Summary of clinical and molecular findings in all the affected individuals with pathogenic variants in *EXOC6B*

	Present study		Campos-Xavier et al. (2018)		Girisha et al. (2016)	
	Family 1	Family 2	Family 3		Family 4	
	Patient 1	Patient 2	Patient 3	Patient 4	Patient 5	Patient 6
General characteristics						
Age (years, at admission)	3	13	12	9.5	20	14
Gender	Male	Female	Female	Female	Male	Male
Consanguinity	−	+	−	−	+	+
Ethnicity	Asian Indian	Turkish	NA	NA	Asian Indian	Asian Indian
Clinical features						
Prominent forehead	+	−	NA	NA	−	−
Height (SD)	85 cm (−2.8 SD)	116 cm (−5.83 SD)	140 cm (−1.6 SD)	125 cm (−1.81 SD)	120 cm (−8 SD)	116 cm (—6SD)
Short neck	+	+	NA	NA	+	+
Barrel-shaped chest	+	+	NA	NA	−	−
Tapering fingers	+	+	+	+	+	+
Genu valgum	+ (bilateral)	+	+	+	+	+
Joint laxity	+	+	NA	NA	+	+
Joint dislocations	+	+	+	+	+	+
Pes planus	+	+	NA	−	+	+
Short toes	+	+	NA	NA	−	−
Other		Hydrocephalus, developmental delay, moderate restriction of pulmonary function, deep palmar crease, high forehead				
Radiological features						
Slender ribs	+	+	NA	NA	+	+
Irregular vertebral end plates	+	+	NA	NA	+	+
Delayed carpal bone ossification	+	+	NA	NA	Absent for proximal row and delayed for distal row; small carpal bones	
Gracile metacarpals and metatarsals	+	+	NA	NA	+	+
Gracile phalanges	+	+	NA	NA	+	+
Scoliosis	−	+	+	+	+	+
Lordosis	+	+	NA	NA	−	−
Kyphosis	−	+	+	+	−	−
Narrow interpedicular distance	+	+	NA	NA	+	+
Epiphyseal dysplasia	+	+	+	+	+	+
Metaphyseal dysplasia	+	+	+	+	+	+
Prominent distal phalangeal tufts	+	+	NA	NA	+	+
Other		Brachycephaly, hydrocephaly, hypoplasia of mesencephalon, thin corpus callosum				
Molecular testing details						
Variants in EXOC6B (NM_015189.3)	c.2122+15447 2197_59588del	c.401T>G	c.915+20070_2197-135947del	c.915+20070_2197-135947del	c.906T>A	c.906T>A
Protein change	p.(GIn708Profs*16)	p.(Leu134Ter)	p.(Gly305_GIn732del)	p.(Gly305_GIn732del)	p.(Tyr302*)	p.(Tyr302*)
Location	Introns 19–20	Exon 4	Exon 9-20	Exons 9-20	Exon 9	Exon 9

Abbreviations: +, Present; -, absent; NA, not available.
