# Supplementary material for: Biallelic loss-of-function variants in EXOC6B are associated with impaired primary ciliogenesis and cause spondylo-epi-metaphyseal dysplasia with joint laxity type 3
Source: Hum Mutat. Author manuscript; Available in PMC 2024 Apr 25. (PMC7615863; doi:10.1002/humu.24478)
Supplement: Supplementary file [file EMS195523-supplement-Supplementary_file.pdf]

Supplementary materials

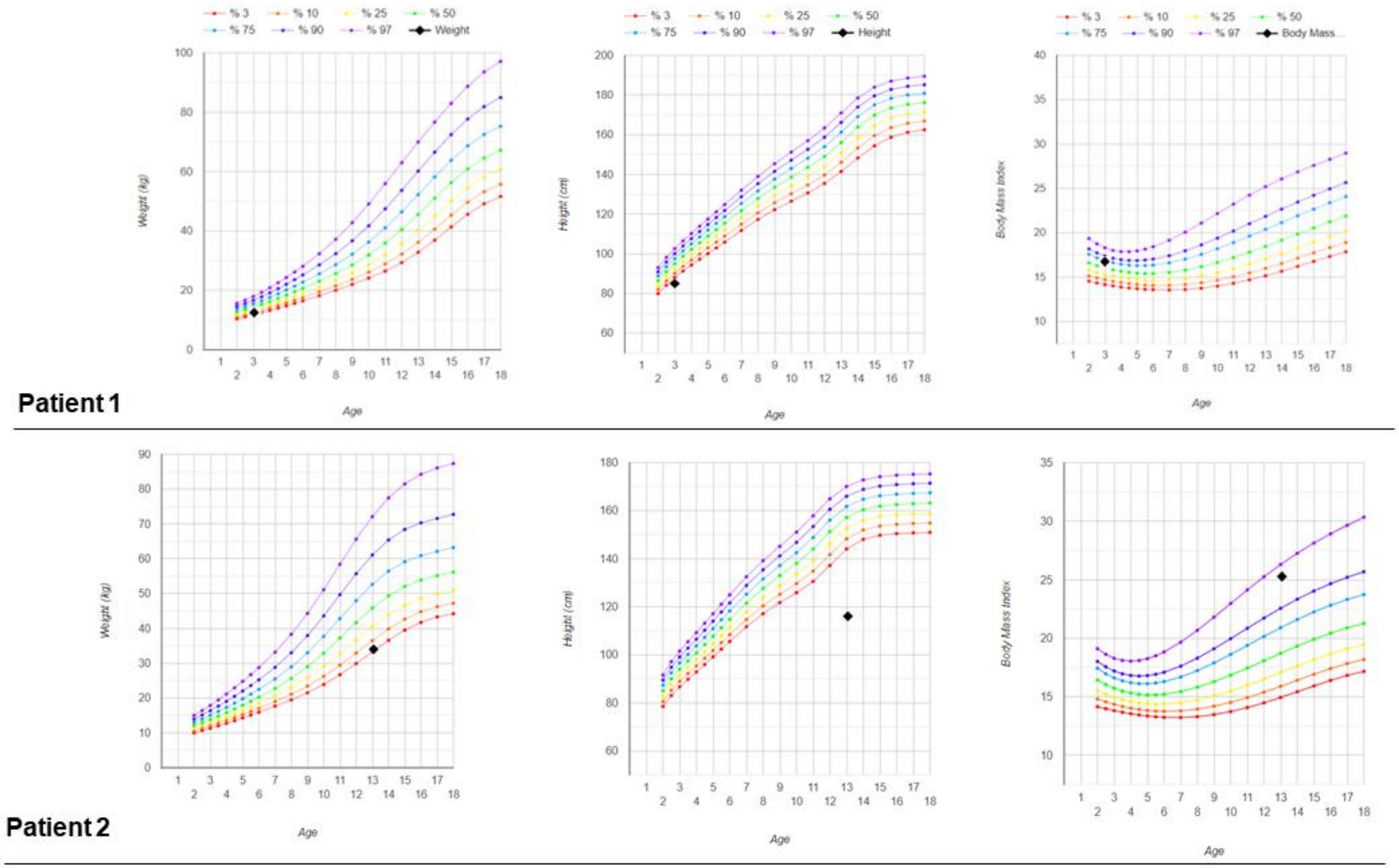

Supp. Figure S1. Weight, height and body mass index charts of Patient 1 (at 3 years of age) and Patient 2 (at 13 years of age) are shown in Table 1 (For anthropometric assessment, Centre for Disease Control measurements are used as a reference).

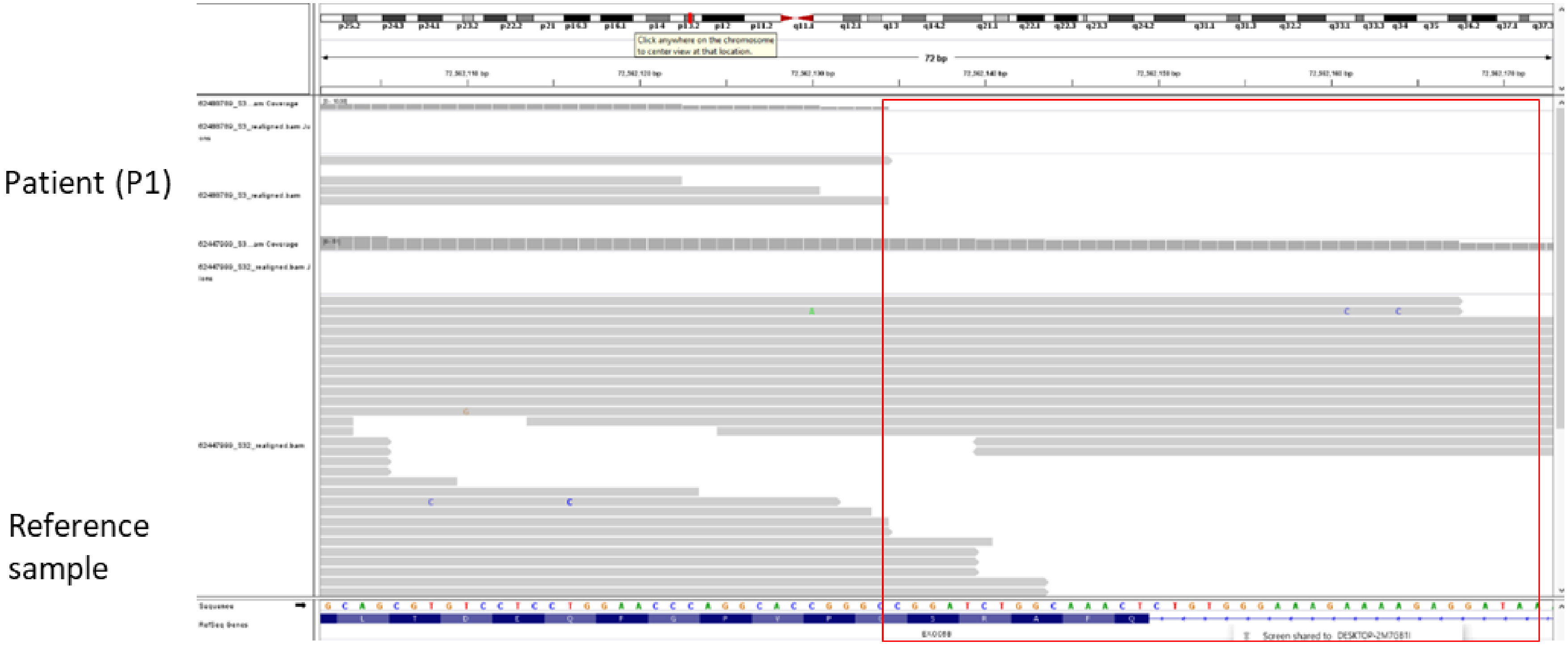

Supp. Figure S2. Integrative Genomics Viewer (IGV) screenshot of patient's genomic location in exon 20 of EXOC6B against a reference sample shows comparatively low number of reads indicating deletion of the region.

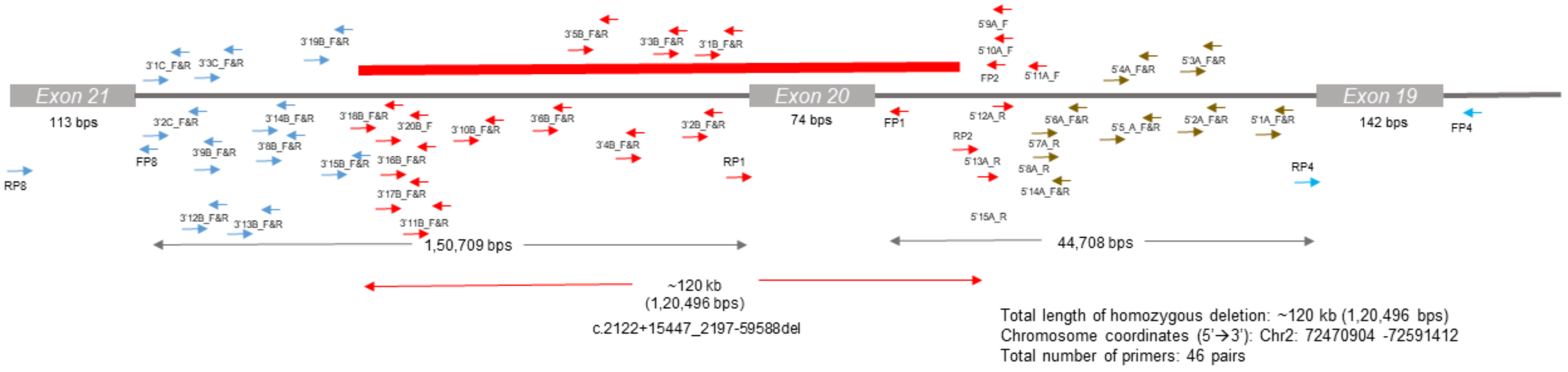

Supp. Figure S3. Primer map representation to find the breakpoint of homozygous deletion in the genomic region of exon 19-21 in EXOC6B (NC_000002.12). Using the set of 46 primers, the complete length of deletion was estimated to be 120496 bps, c.2122+15447_2197-59588del (chr2:72470904-72591411).

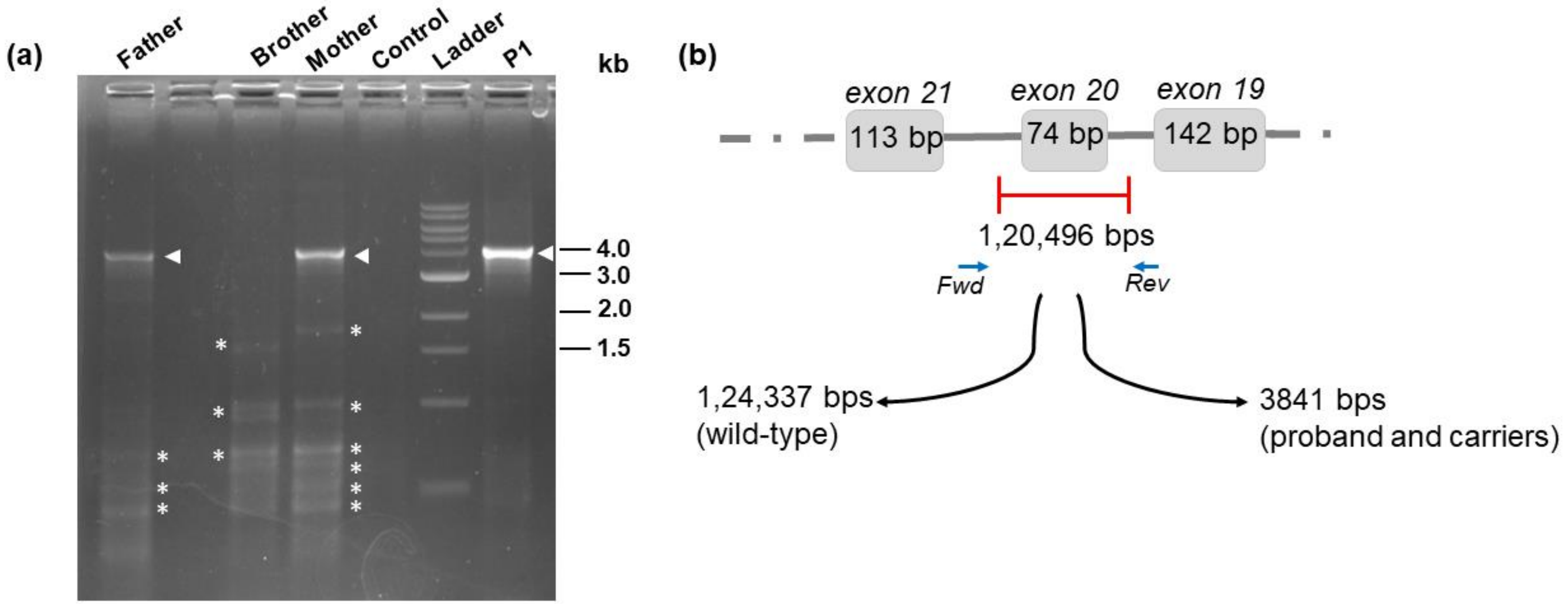

Supp. Figure S4. Agarose gel image for segregation analysis of the homozygous deletion c.2122+15447_2197-59588del in EXOC6B in family 1. (a) PCR with genomic DNA using primers represented in blue that are immediately flanking the deleted region showed an amplicon of 3,841 bp (white arrowhead) in the proband (P1) and her parents (heterozygous carriers). This primer pair would yield an amplicon of 1,24,337 bp that is expectedly not detectable in her brother and an unrelated control (wild-type). Non-specific bands are indicated by white asterisks. (b) Schematic representation of segregation of (a).

Term name	Term ID	p_{adj}	$-\log_{10}(p_{adj})$
extracellular matrix structural constituent	GO:0005201	2.007×10^{-11}	
structural molecule activity	GO:0005198	1.914×10^{-8}	
collagen binding	GO:0005518	4.123×10^{-7}	
protein binding	GO:0005515	4.236×10^{-5}	

Term name	Term ID	p_{adj}	$-\log_{10}(p_{adj})$
extracellular matrix organization	GO:0030198	6.159×10^{-12}	
extracellular structure organization	GO:0043062	6.578×10^{-12}	
external encapsulating structure organization	GO:0045229	7.498×10^{-12}	
cellular component organization	GO:0016043	1.033×10^{-10}	
cellular component organization or biogenesis	GO:0071840	7.081×10^{-10}	

Supp. Figure S5. RNA sequencing in fibroblast sample of Patient 2 revealed that pathways related with extracellular matrix were decreased.

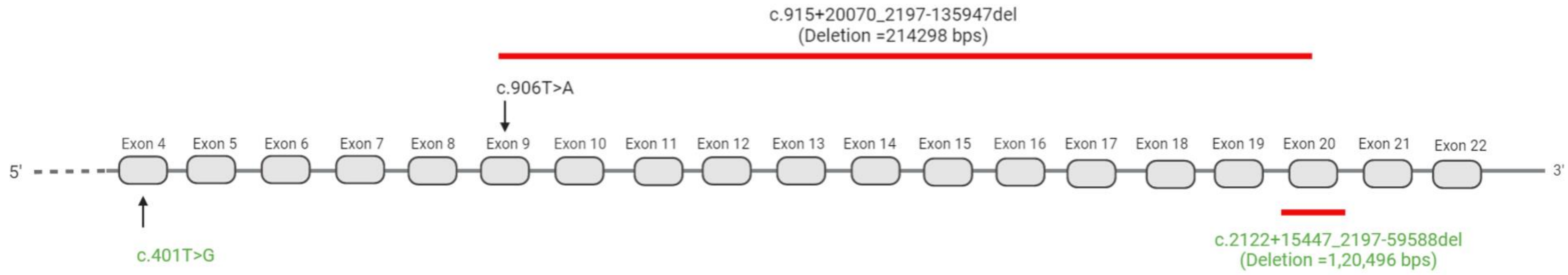

Supp. Figure S6. Schematic representation of EXOC6B gene structure demonstrating positions of the variants causing spondylo-epi-metaphyseal dysplasia with joint laxity, type 3 along with the variants identified in the present study (green).

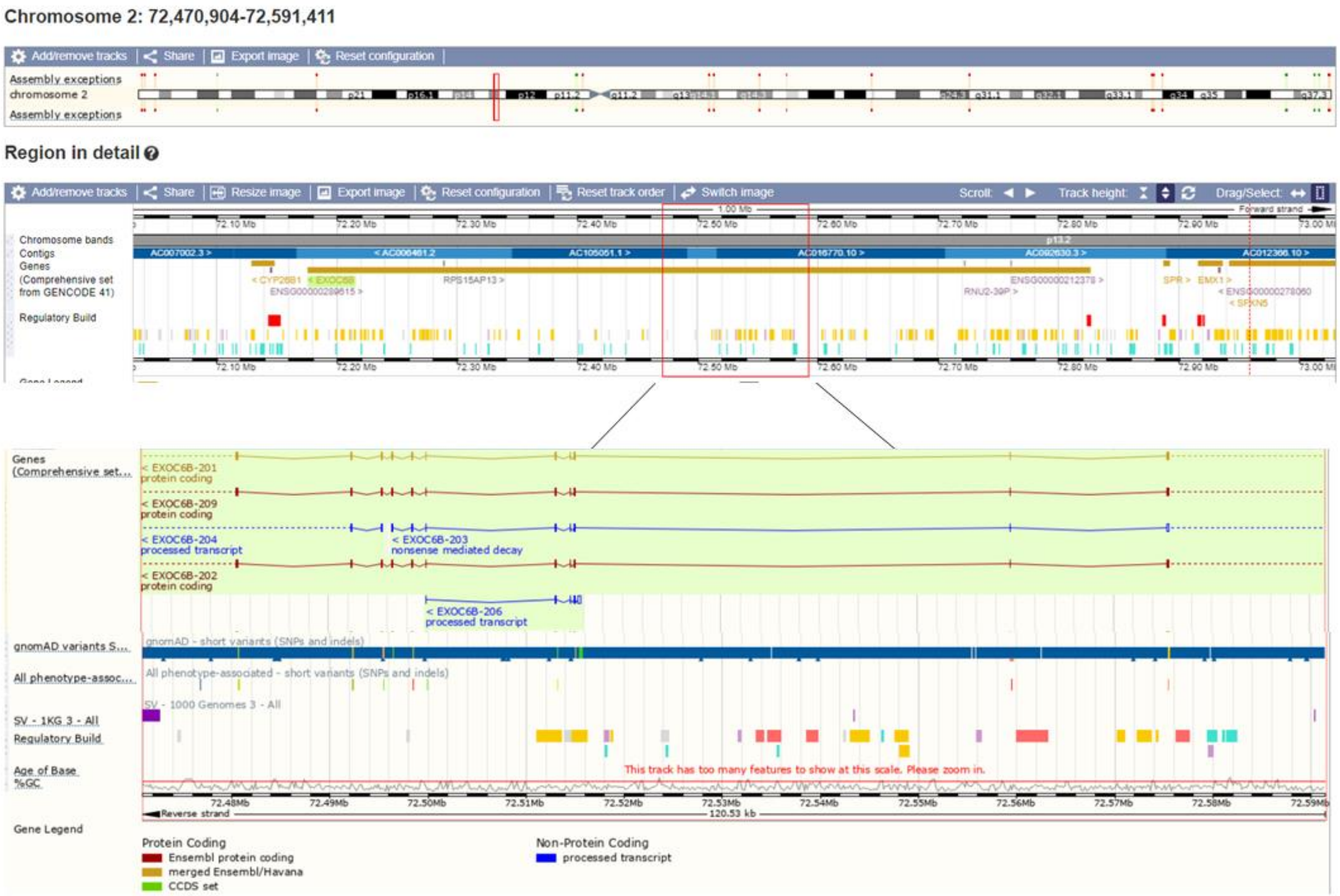

Supp. Figure S7. Region viewer tool from ensemble (https://asia.ensembl.org/index.html) shows essential regulatory components in the deleted chromosomal region (chr 2: 72470904-72591411) of EXOC6B. Link for reference: https://asia.ensembl.org/Homo_sapiens/Location/View?db=core;g=ENSG00000144036;r=2:72470904-72591411.

Suppl. Table S1. Filtering strategy used for variant analysis for P1 from family 1

	P1	
Total called variants	38,452	
Minor allele frequency (<=1%)*	8,708	
Rare variants in In house database (<=1% in in-house data of 1455 exomes of Indian ethnicity)	3,918	
Exonic and canonical splicing variants	959	
Biallelic variants not present in gnomAD v2.1.1	89	
Number of variants with known OMIM phenotype	Homozygous	7
	Compound heterozygous	24
Pathogenic copy number variant correlating with the observed phenotype	1(EXOC6B-p.(Gln708_Gln732del))	

*Frequency less than or equal to 1% in population databases (gnomAD v2.1.1 and ExAC).

Suppl. Table S1 (continued). Filtering strategy used for variant analysis for P2 from family 2

	P2
Total called variants	44,796
Minor allele frequency (<=1%)	2,324
Biallelic variants not present in gnomAD v2.1.1	257
Exonic and canonical splicing variants	85
Probably pathogenic variants in homozygous regions	8
Probably pathogenic variants correlating with the observed phenotype	1 (EXOC6B p.(Leu134Ter))

Suppl. Table S2. List of RT-PCR and RT-qPCR primer sequences used for family 1

		Primer sequence (5'-3')	Product size (bp)
GAPDH	Forward	ACCTGCCAAATATGATGAC	192
	Reverse	TCATACCAGGAAATGAGCTT	
***EXOC6B* set 1**	Forward	ACCTTGGGAGCATTACAGCA	194
	Reverse	TACTTGCAGTTGGGCTGACC	
***EXOC6B* set 2**	Forward	ACTCCGTACCCACTCGTCTT	178
	Reverse	ACCTGCCCACTTCTTTAGCA	
***EXOC6B* set 3**	Forward	AACAGAGGCGAAAACAGGCT	184
	Reverse	AGTGCCATTTCCCACAGTTCA	
***EXOC6B* set 4**	Forward	CCCACGCTCAGGTCTGTTTA	2195
	Reverse	GTCAGAGCAGTCACTGGGTT	
***EXOC6B* set 5**	Forward	ACTCCGTACCCACTCGTCTT	1146
	Reverse	GTCAGAGCAGTCACTGGGTT	

Suppl. Table S2 (continued). List of RT-PCR and RT-qPCR primer sequences used for family 2

		Primer sequence (5'-3')	Product size (bp)

ACTB	Forward	CGCAAAGACCTGTACGCCAAC	164
	Reverse	GAGCCGCCGATCCACACG	
EXOC6B	Forward	CTGTGTCTTCCAGTCCTAGAG	156
	Reverse	GATGTTGTCCACCATCACC	

Supp. Table S3. Result of copy number variant analysis in patient 1 (P1) using ExomeDepth tool (Total number of CNVs (n=53, 31 deletions and 22 duplications detected in P1 from Family 1).

start.p	end.p	type	nexons	start	end	chromosome	id	BF	reads.expected	reads.observed	reads.ratio	Conrad.hg19	exons.hg19
470	471	deletion	2	1848414	1848632	1	chr1:1848414-1848632	7.2	191	109	0.571	NA	CALML6_5,CALML6_6
1180	1183	duplication	4	9098888	9100248	1	chr1:9098888-9100248	6.76	599	799	1.33	NA	SLC2A5_9,SLC2A5_8,SLC2A5_7, SLC2A5_6
2222	2223	deletion	2	17316383	17316782	1	chr1:17316383-17316782	5.9	283	173	0.611	NA	ATP13A2_22,ATP13A2_21
11169	11173	duplication	5	1.46E+08	1.46E+08	1	chr1:146400092-146405926	11.7	40	127	3.18	NA	NBPF12_9,NBPF12_10,NBPF12_11, NBPF12_12,NBPF12_13
12797	12797	deletion	1	1.55E+08	1.55E+08	1	chr1:155282047-155282186	4.78	102	50	0.49	NA	FDPS_4
23958	23958	deletion	1	72562077	72562149	2	chr2:72562077-72562149	7.19	39	3	0.0769	NA	EXOC6B_20
25590	25597	duplication	8	99778422	99797444	2	chr2:99778422-99797444	7.7	561	755	1.35	NA	LIPT1_4,MITD1_7,MITD1_6, MITD1_5,MITD1_4,MITD1_3, MITD1_2,MITD1_1
34150	34151	deletion	2	2.42E+08	2.42E+08	2	chr2:241631332-241633966	5.58	300	178	0.593	NA	AQP12A_1,AQP12A_2,AQP12A_3
46667	46675	deletion	9	9174900	9237700	4	chr4:9174900-9237700	24.1	136	10	0.0735	NA	FAM90A26P_4,FAM90A26P_5, FAM90A26P_6,FAM90A26P_7, USP17L13_1,USP17L10_1, USP17L11_1,USP17L12_1, USP17L13_2,USP17L15_1
60876	60877	duplication	2	1.75E+08	1.75E+08	5	chr5:175388055-175388437	6.63	66	131	1.98	NA	THOC3_5,THOC3_4

65608	65609	deletion	2	43484849	43485115	6	chr6:43484849-43485115	5	326	209	0.641	NA	POLR1C_1,POLR1C_2
71030	71030	deletion	1	1.68E+08	1.68E+08	6	chr6:167790035-167790192	7.77	53	9	0.17	NA	TCP10_5
71773	71774	duplication	2	6000323	6004201	7	chr7:6000323-6004201	9.64	39	130	3.33	NA	RSPH10B_5,RSPH10B_4
71939	71944	duplication	6	6836200	6844686	7	chr7:6836200-6844686	22.5	120	314	2.62	NA	RSPH10B2_19,RSPH10B2_20, CCZ1B_15,CCZ1B_14,CCZ1B_13, CCZ1B_12
74600	74602	deletion	3	74234500	74237335	7	chr7:74234500-74237335	7.63	82	33	0.402	NA	GTF2IRD2_7,GTF2IRD2_6, GTF2IRD2_5
74621	74622	duplication	2	74479108	74480461	7	chr7:74479108-74480461	6.59	27	81	3	NA	WBSCR16_5,WBSCR16_4
79463	79463	deletion	1	1.49E+08	1.49E+08	7	chr7:149174014-149174138	4.64	83	39	0.47	NA	ZNF746_6
86350	86352	deletion	3	1.45E+08	1.45E+08	8	chr8:145438619-145439211	11.9	55	0	0	NA	FAM203B_2,FAM203B_3, FAM203B_4
88526	88532	deletion	7	40701711	40784603	9	chr9:40701711-40784603	24.1	1919	1200	0.625	NA	SPATA31A3_2,SPATA31A3_3, SPATA31A3_4,FAM74A3_1, ZNF658_5,ZNF658_4,ZNF658_3
88600	88611	duplication	12	43861004	43920370	9	chr9:43861004-43920370	12.7	187	308	1.65	NA	CNTNAP3B_13,CNTNAP3B_14, CNTNAP3B_15,CNTNAP3B_16, CNTNAP3B_17,CNTNAP3B_18, CNTNAP3B_19,CNTNAP3B_20, CNTNAP3B_21,CNTNAP3B_22, CNTNAP3B_23,CNTNAP3B_24
88639	88657	deletion	19	67926874	67987998	9	chr9:67926874-67987998	15.7	163	63	0.387	NA	ANKRD20A1_1,ANKRD20A1_2, ANKRD20A1_3,ANKRD20A1_4, ANKRD20A1_5,ANKRD20A1_6, ANKRD20A1_7,ANKRD20A1_8, ANKRD20A1_9,ANKRD20A1_10, ANKRD20A1_11,ANKRD20A1_12, ANKRD20A1_13,ANKRD20A1_14, ANKRD20A1_15,RP11-195B21.3_3, RP11-195B21.3_4,RP11-195B21.3_5, RP11-195B21.3_6
88712	88726	deletion	15	70862530	70919121	9	chr9:70862530-70919121	16.7	314	164	0.522	NA	CBWD3_3,CBWD3_4,AL353608.1_1, CBWD3_5,CBWD3_6,CBWD3_7, CBWD3_8,CBWD3_9,CBWD3_10, CBWD3_11,CBWD3_12,CBWD3_13, CBWD3_14,CBWD3_15,FOXD4L3_1
91582	91582	deletion	1	1.24E+08	1.24E+08	9	chr9:124093667-124093726	5.17	82	36	0.439	NA	GSN_16
94612	94612	deletion	1	5037512	5037676	10	chr10:5037512-5037676	5.36	118	57	0.483	NA	AKR1C2_7

95350	95359	deletion	10	17865074	17905692	10	chr10:17865074-17905692	13.2	542	331	0.611	NA	MRC1L1_2,MRC1L1_3,MRC1L1_4,MRC1L1_5,MRC1L1_6,MRC1L1_7,MRC1L1_8,MRC1L1_9,MRC1L1_10,MRC1L1_11
95384	95394	duplication	11	18112045	18155690	10	chr10:18112045-18155690	29.8	156	415	2.66	NA	MRC1_2,MRC1_3,MRC1_4,MRC1_5,MRC1_6,MRC1_7,MRC1_8,MRC1_9,MRC1_10,MRC1_11,MRC1_12
96454	96461	deletion	8	46312644	46342795	10	chr10:46312644-46342795	35.7	397	84	0.212	NA	FAM25E_1,AGAP4_7,AGAP4_6,AGAP4_5,AGAP4_4,AGAP4_3,AGAP4_2,AGAP4_1
103289	103290	deletion	2	5270599	5271034	11	chr11:5270599-5271034	12	64	4	0.0625	NA	HBG1_2,HBG1_1
105808	105808	deletion	1	47368179	47368195	11	chr11:47368179-47368195	5.36	64	24	0.375	NA	MYBPC3_11
113850	113851	deletion	2	6959632	6960116	12	chr12:6959632-6960116	6.97	392	239	0.61	NA	CDCA3_3,U47924.25_3,CDCA3_2,U47924.25_2
114468	114469	duplication	2	11034835	11035297	12	chr12:11034835-11035297	5.45	232	338	1.46	NA	PRH1_4,PRH1_3
116160	116162	deletion	3	48131351	48132030	12	chr12:48131351-48132030	6.31	205	123	0.6	NA	RAPGEF3_28,RAPGEF3_27,RAPGEF3_26
116240	116242	duplication	3	48370596	48371210	12	chr12:48370596-48371210	5.58	796	1051	1.32	NA	COL2A1_48,COL2A1_47,COL2A1_46
124178	124178	deletion	1	24468279	24468340	13	chr13:24468279-24468340	12	163	50	0.307	NA	C1QTNF9B_2
128049	128050	deletion	2	23790680	23791504	14	chr14:23790680-23791504	9.04	252	133	0.528	NA	PABPN1_1,AL049829.1_2,BCL2L2-PABPN1_3,PABPN1_2,AL049829.1_1
133670	133670	duplication	1	23690458	23690585	15	chr15:23690458-23690585	5.57	23	71	3.09	NA	GOLGA6L2_2
133990	134001	duplication	12	30429070	30434585	15	chr15:30429070-30434585	8.99	334	467	1.4	NA	GOLGA8T_2,GOLGA8T_3,GOLGA8T_4,GOLGA8T_5,GOLGA8T_6,GOLGA8T_7,GOLGA8T_8,GOLGA8T_9,GOLGA8T_10,GOLGA8T_11,GOLGA8T_12,GOLGA8T_13
134160	134162	duplication	3	32685495	32686434	15	chr15:32685495-32686434	5.65	129	198	1.53	NA	GOLGA8K_17,GOLGA8K_16,GOLGA8K_15
134204	134216	duplication	13	32890090	32896179	15	chr15:32890090-32896179	9.36	475	662	1.39	NA	GOLGA8N_7,GOLGA8N_8,GOLGA8N_9,GOLGA8N_10,GOLGA8N_11,GOLGA8N_12,GOLGA8N_13,GOLGA8N_14,GOLGA8N_15,GOLGA8N_16,GOLGA8N_17,GOLGA8N_18,GOLGA8N_19

141612	141613	deletion	2	2569220	2569746	16	chr16:2569220-2569746	5.12	468	307	0.656	NA	TBC1D24_2,ATP6V0C_2,ATP6V0C_3
147618	147620	duplication	3	75239232	75239890	16	chr16:75239232-75239890	8.17	132	226	1.71	NA	CTRB2_5,CTRB2_4,CTRB2_3
151380	151382	duplication	3	14139174	14140150	17	chr17:14139174-14140150	5.81	515	689	1.34	NA	CDRT15_3,CDRT15_2,CDRT15_1
160271	160271	deletion	1	2656076	2656260	18	chr18:2656076-2656260	5.33	150	77	0.513	NA	SMCHD1_1
165690	165691	deletion	2	10108661	10108825	19	chr19:10108661-10108825	5.97	192	111	0.578	NA	COL5A3_10,COL5A3_9
165731	165731	deletion	1	10222399	10222416	19	chr19:10222399-10222416	5.86	122	57	0.467	NA	P2RY11_1
165786	165788	deletion	3	10362931	10363377	19	chr19:10362931-10363377	10.4	403	245	0.608	NA	MRPL4_1,MRPL4_2,MRPL4_3
166916	166917	duplication	2	14508471	14508747	19	chr19:14508471-14508747	6.61	106	182	1.72	NA	CD97_7,CD97_8
171745	171746	duplication	2	48259786	48260001	19	chr19:48259786-48260001	5.18	289	407	1.41	NA	GLTSCR2_11,GLTSCR2_12
181694	181722	duplication	29	22311487	22569660	22	chr22:22311487-22569660	53.4	5867	7898	1.35	NA	TOP3B_18,TOP3B_17,TOP3B_16, TOP3B_15,TOP3B_14,TOP3B_13, TOP3B_12,TOP3B_11,TOP3B_10, TOP3B_9,TOP3B_8,TOP3B_7 ,TOP3B_6,TOP3B_5,TOP3B_4, TOP3B_3,TOP3B_2,IGLV4-69_1, IGLV4-69_2,IGLV8-61_1,IGLV8-61_2, IGLV4-60_1,IGLV4-60_2,IGLV6-57_1, IGLV6-57_2,IGLV11-55_1, IGLV11-55_2,IGLV10-54_1, IGLV10-54_2
182121	182123	duplication	3	25428577	25445331	22	chr22:25428577-25445331	6.25	77	139	1.81	NA	KIAA1671_2,KIAA1671_3, KIAA1671_4
183133	183134	duplication	2	36684299	36684985	22	chr22:36684299-36684985	5.12	278	394	1.42	NA	MYH9_34,MYH9_33
184267	184267	deletion	1	43606050	43606246	22	chr22:43606050-43606246	5.55	142	71	0.5	NA	SCUBE1_19
185004	185007	deletion	4	50945068	50946874	22	chr22:50945068-50946874	7.41	541	353	0.652	NA	LMF2_3,LMF2_2,LMF2_1,NCAPH2_1

In-silico prediction tools

ClinGen CNV Pathogenicity Calculator (https://cnvcalc.clinicalgenome.org/cnvcalc/cnv-loss) was used for calculating the pathogenicity score for the copy number variant (deletion) observed in patient 1 (P1) via Exome depth tool in the present study. This tool utilizes the CNV scoring metrics that appear in the ACMG Technical Standards (Riggs et al., 2020). The scoring metric suggested that the c.2122+15447_2197-59588del copy number variant in *EXOC6B* is a pathogenic variant, with a score of 1.45.

For the disease-causing variant observed in patient 2 (P2), c.401T>G, multiple *in-silico* prediction tools were used. Based on the scoring metrics of Mutation taster (score: 1; Deleterious), BayesDel (score: 0.66; Deleterious), and GERP (score: 5.91; Uncertain) score the null variant was classified as pathogenic following the ACMG-AMP guidelines (Richards et al., 2015).

Supporting References

Richards, S., Aziz, N., Bale, S., Bick, D., Das, S., Gastier-Foster, J., … Rehm, H. L. (2015). Standards and guidelines for the interpretation of sequence variants: A joint consensus recommendation of the American College of Medical Genetics and Genomics and the Association for Molecular Pathology. *Genetics in Medicine*, *17*(5), 405–423. doi: 10.1038/gim.2015.30

Riggs, E. R., Andersen, E. F., Cherry, A. M., Kantarci, S., Kearney, H., Patel, A., … Martin, C. L. (2020). Technical standards for the interpretation and reporting of constitutional copy-number variants: A joint consensus recommendation of the American College of Medical Genetics and Genomics (ACMG) and the Clinical Genome Resource (ClinGen). *Genetics in Medicine*, *22*(2), 245–257. doi: 10.1038/s41436-019-0686-8
